# Folate biofortification strategy by elicitation in kale baby leaves

**DOI:** 10.3389/fpls.2026.1737335

**Published:** 2026-04-07

**Authors:** Anna Bonasia, Corrado Lazzizera, Giuseppina Spadaccino, Francesco Di Nunzio, Luigi Giuseppe Duri, Maurizio Quinto, Antonio Elia, Giulia Conversa

**Affiliations:** Department of Agriculture, Food, Natural Resources and Engineering (D.A.F.N.E.), University of Study of Foggia, Foggia, Italy

**Keywords:** 5-formyl-tetra-hydro-folate, 5-methyl-tetra-hydro-folate, antioxidant compounds, elicitors, nitrate, phenylalanine, salicylic acid

## Abstract

**Introduction:**

Humans cannot synthesize folates, so they must be acquired through dietary sources, with plants serving as a primary source. To enhance folate levels in vegetables, this study investigated the pre-harvest application of elicitors on kale (‘Cavolo Nero’) baby leaves under greenhouse-autumn conditions in Southern Italy.

**Methods:**

Phenylalanine (0.37 µM) and salicylic acid (250 µM) were applied three times and compared to a water control. Folate content, alongside biometric traits (yield, leaf area and number, dry matter), bio-physiological parameters (chlorophyll content; measurements of gas exchange and chlorophyll *a* fluorescence), and compositional properties (nitrate, vitamin C, phenols, flavonoids, carotenoids, antioxidant capacity) were determined.

**Results:**

The treatments maintained yield (1.0 kg m^-2^, on average), while both elicitors increased the folate concentration (1,283 µg 100 g^-1^ d.w., on average) compared to the control (1,203 µg 100 g^-1^ d.w.). Salicylic acid application enhanced antioxidant compounds (vitamin C, carotenoids) without modifying antioxidant capacity and nitrate levels. In contrast, phenylalanine decreased antioxidant compounds (phenols, vitamin C) along with antioxidant capacity, and enhanced nitrate level (+56%).

**Discussion:**

Considering the limited literature available on this topic, the present work provides an important first step toward producing folate-biofortified baby leaf vegetables with enhanced beneficial traits.

## 1 Introduction

Humans cannot synthesize folates (a collective term for folic acid and its derivatives, also known as vitamin B9 or folacin) and are entirely dependent on dietary sources to meet their folate requirements, classifying it as an essential nutrient.

Folates play a critical role in human health, particularly for women during pregnancy, as adequate intake reduces the risk of neural tube defects (NTD) as anencephaly (a severe and fatal NTD where a baby is born without major parts of the brain, skull, and scalp) and *spina bifida* (a NTD occurring when a baby’s spine and spinal cord do not develop properly during the first month of pregnancy in newborns) (Imbard et al., 2013).

Folate deficiency can arise from multiple factors, including inadequate diet intake, congenital disorders, malabsorption conditions (e.g., celiac disease, hemolytic anemia, and dialysis), and other health-related issues (e.g., alcoholism, pregnancy, and lactation). Clinically, folate deficiency is associated with a range of physical symptoms (anorexia, nausea, vomiting, abdominal pain, diarrhea), neuropsychiatric manifestations (cognitive impairment, dementia, depression), and severe health complications (anemia, increased stroke risk, and prostate cancer) (Bo et al., 2020). Notably, individuals with celiac disease often experience impaired folate absorption, leading to lower average daily intake compared to the general population (Unalp-Arida et al., 2022). Emerging epidemiological evidence further supports the protective role of dietary folate. A recent population-based cohort study in Europe suggests that higher folate intake from food sources may be associated with a reduced risk of pancreatic cancer (Larsson et al., 2006).

Folates are synthesized *de novo* in bacteria, fungi, and plants. The composition of folates in plants includes di-hydro-folates and tetra-hydro-folates; of the latter, 5-methyl-H4-folate (5-m-THF) and 5-formyl-H4-folate (5-f-THF) are the most representative (Kołton et al., 2022). Their concentration in plants varies significantly depending on genotype, both between (Gorelova et al., 2017) and within species (Nascimento et al., 2022). Staple crops such as rice, maize, wheat, potatoes and other starchy foods contain relatively low folate levels (0.1-1.0 nmol g^-1^ edible portion, e.p.). In contrast, dark green leafy vegetables, including spinach, kale, romaine lettuce, broccoli, and asparagus, are considered good sources (5.0 nmol g^-1^ e.p.), while legumes rank among the richest dietary sources (9.0-10.0 nmol g^-1^ e.p.) (Rébeillé et al., 2006). Beyond genetic factors, folate biosynthesis in plants is also regulated by other factors, such as growth stage and growing conditions (Gorelova et al., 2017).

Folate biofortification has been deeply managed via breeding or metabolic engineering towards several target crops (or tissues), as widely reviewed by Strobbe and Van Der Straeten (2017). To enhance folate levels in crops, metabolic engineering via genetic modification has been explored in model plants *Arabidopsis thaliana* (Hossain et al., 2004), staple crops such as rice (Storozhenko et al., 2007), and vegetables such as tomato (Díaz De La Garza et al., 2007) and lettuce (Nunes et al., 2009). However, due to regulatory and ethical concerns surrounding transgenic approaches, non-genetically modified organism (GMO) strategies like agronomic biofortification have gained attention, including elicitation (Saini et al., 2016). Elicitation involves applying specific stressors, or elicitors, to stimulate natural plant defense mechanisms, which in turn improve both nutritional content and functional properties.

Few studies have explored agronomic folate biofortification in vegetables via elicitor application, such as phytohormones or organic acids (Puthusseri et al., 2012; Puthusseri et al., 2013; Watanabe et al., 2017). Notably, only Puthusseri et al. (2013) investigated the use of preharvest foliar sprays.

In *callus* cultures of coriander treated with methyl-jasmonate (phyto-hormone) and salicylic acid (organic acid) elicitors, a rapid 2-fold increase of folates occurred, particularly in response to salicylic acid (Puthusseri et al., 2012). Foliar-sprayed salicylic acid doubled folate concentration in greenhouse-grown coriander (Puthusseri et al., 2013). Soilless-grown spinach treated with phenylalanine (amino acid) via nutrient solution exhibited a two-fold increase in folates (Watanabe et al., 2017). Phenylalanine was also investigated by Teixeira et al. (2018) to evaluate its effects on nitrogen metabolism in soybeans, comparing different application methods (foliar spray and seed treatment) and cultivation systems (greenhouse and open field).

For salicylic acid-mediated elicitor effects, potential mechanisms include up-regulation of folate biosynthetic genes, suppression of folate degradation pathways, enhanced antioxidant production, and/or modulation of folate-binding proteins (Scott et al., 2000). Regarding phenylalanine-mediated enrichment, a redirecting of the chorismate mutase activity to para-amino-benzoate (p-ABA, the folate precursor) synthesis occurs, instead of chorismate synthesis (Basset et al., 2004).

Given the dietary importance of folate and the limited research on the agronomic biofortification of vegetables, this study aimed to evaluate as folate elicitors the efficacy of two distinct classes of compounds: salicylic acid (an organic acid) and phenylalanine (an amino acid) via foliar application. Due to the scarcity of available information, we investigated the potential of these compounds using a single concentration informed by previous literature, notwithstanding the known importance of dose-response dynamics.

We chose baby leaves, a popular category of immature leafy greens consumed raw in salads, due to their high nutrient density and aesthetic appeal (Raffo and Paoletti, 2022). As they are typically consumed raw and preserve nutrient density, they represent an ideal target for folate enrichment, with the widely cultivated Brassicaceae family being a particularly prominent choice.

To this end, a greenhouse experiment was conducted on baby kale - *Brassica oleracea* (Acephala Group) - leaves to evaluate the effect of these elicitors on folate concentration and profile. Additionally, biometric and physiological traits were monitored to ensure that elicitor application did not negatively impact plant development or metabolic efficiency. Given the high nutritional density of baby-leaf crops, the study also evaluated the comprehensive nutritional profile of kale baby leaves by quantifying health-promoting phytochemicals (phytosterols, flavonoids, anthocyanins, vitamin C, and carotenoids) alongside anti-nutritional factors, specifically nitrates.

## 2 Materials and methods

### 2.1 Plant material and growing conditions

The trial was conducted under autumn conditions in a plastic greenhouse at the commercial farm Ortogourmet in Mola di Bari (BA), southern Italy (41°02′16.3″ N 17°06′20.3″ E, 5 m a.s.l.). The mean maximum and mean minimum temperatures of the cultivation period (5 November-12 December 2022) were 22.6 and 10.9 °C, respectively.

The ‘Cavolo Nero’ (kale) *Brassica oleracea* (Acephala Group) was grown as a baby leaf. Kale seeds (De Corato Sementi, Andria, BT, Italy) were manually distributed on the substrate surface at a density of 1,500 seeds m^-2^ in grow-box trays (23x48x6 cm, W×L×D, 0.110 m^2^) filled with peat (Brill 3 TYPical, Gebr. Brill Substrate GmbH & Co. KG, Georgsdorf, NI, Germany). During the first 4 days, the trays were placed in a germination chamber under dark conditions at 20 °C. On day five, seedlings were exposed inside the greenhouse, from 8 to 5 mol m^-^² day^-1^ as Photosynthetic Photon Flux Density (PPFD). Upon emergence, trays were arranged on aluminium benches (420*180 cm), and an ebb and flow irrigation system was adopted. Trays were irrigated daily with rainwater until germination was complete. After germination, a half-strength Hoagland nutrient solution was used. It was prepared by mixing macro- and micro-nutrients with rainwater, resulting in a final concentration (mg L^−1^) of 112 N, 120 K, 80 Ca, 31 P, 16 S, 12 Mg, 0.135 B, 0.56 Fe, 0.055 Mn, 0.0655 Zn, 0.016 Cu, and 0.025 Mo. A fertilizer dosing system (Priva NutriFit, De Lier, Netherlands) was employed.

The elicitors applied were (a) salicylic acid (SA), 250 µM (Puthusseri et al., 2013), and (b) phenyl-alanine (PA), 0.37 µM (derived from 30 mg ha^-1^) (Sigma-Aldrich Merck KGaA, Darmstadt, Germany) (**Supplementary Figure 1**). The phenylalanine concentration was based on the work of Teixeira et al. (2018), which represents the only study to date defining a suitable dose (30 mg ha^-^¹) for foliar application. The treatments were carefully sprayed (50 mL m^-2^) thrice during the cultivation cycle, at 1^st^-2^nd^ and 2^nd^-3^rd^ and 3^rd^-4^th^ true leaf stages, using a hand-sprayer. The untreated control received distilled water. SA was supplemented after dissolving in absolute ethanol, and then it was added dropwise to water, so that ethanol/water was maintained at 1/1000 v/v.

The three treatments were tested in a randomized complete block design (RCBD) with three replicates, where each bench served as a block. The experimental unit (e.u.) consisted of 20 trays and the whole trial of 180 trays. Trays within each bench were rotated daily to ensure uniform environmental conditions. Each tray contained 165 seeds/seedlings at sowing. Elicitor volume sprayed per e.u. was 110 mL, corresponding to 5.5 mL per tray and consistent with the declared application volume (50 mL m^-2^).

Plants were harvested, 37 days after sowing, by manually cutting the leaf bunches about 1 cm above the collar when they reached the optimal stage for fresh consumption as baby leaves (ca. 10 cm).

### 2.2 Measurements

For bio-physical measurements (leaf fresh weight, number and area, leaf color, leaf dry matter and chlorophyll concentration), six trays per each e.u. were used, totalizing n=18 fresh sub-samples per treatment.

Physiological measurements (leaf gas exchange and chlorophyll *a* fluorescence) were assessed on two plants from two trays per e.u. and two fully expanded leaves per plant were used, totalizing n=12 reading sub-samples per treatment.

Folate and nitrate concentration, antioxidant compounds (phenols, anthocyanins, flavonoids, vitamin C, carotenoids, and phytosterols) and antioxidant capacity were assessed on 12 trays per e.u.; specifically, four bulks per e.u. (three trays per bulk) were created; four lyophilized samples per e.u. were used, totalizing n=12 lyophilized sub-samples per treatment.

#### 2.2.1 Bio-physical measurements

The bio-physical traits as leaf fresh weight (yield), number and area, leaf dry matter concentration, and leaf color were determined on fresh material.

In order to determine the dry weight (DW) of leaves, fresh material (FW) was dried in a thermo-ventilated oven at 70 °C until it reached a constant mass. Dry matter concentration (DM) was calculated as DW/FW*100. Leaf area was measured using a LI-COR 3100 (LICOR, Lincoln, NE, USA). The Soil Plant Analysis Development (SPAD) index was measured by a SPAD-502 Chlorophyll meter (Konica Minolta Inc., Tokyo, Japan). Color measurements were acquired in the CIELAB (1976) color space parameters: L* (lightness index), a* (chroma component), and b* (chroma component). A portable tri-stimulus color meter (Minolta Chroma Meter CR-200; Minolta Camera Co. Ltd., Osaka, Japan) was used. Derived parameters such as hue angle [h°=arctan(b*/a*)] and chroma [C*=(a*)^2^+(b*)^2^] indicating hue and color intensity, were calculated.

Chlorophyll concentrations (*a*, *b*, and total) were determined on sample material, previously lyophilized (ScanVac, Chemie s.r.l., Valenzano, Bari, Italy; vacuum less than 0.2 mbar; condenser temperature -54 °C.), mill-ground (IKA, Labortechnik, Staufen, Germany), using the spectrophotometric method described by Sumanta et al. (2014), with minor modifications. Material samples (0.05 g) were mixed with 1.5 mL 95% ethanol (v:v) in a 10 mL screw-cap tube. The mixture underwent ultrasonic treatment using an ultrasonic power cleaner (DU-32 Digital; Argo Lab, Carpi, MO, Italy) at room temperature for 30 minutes. After sonication, samples were centrifuged in a refrigerated centrifuge (Beckman Coulter AllegraTM 25, Fullerton, CA, USA) for 15 min at 4,000 g and 4 °C. The supernatant was collected, and this process was repeated twice. The combined supernatants were filtered using reinforced nylon membrane filters (0.22 μm). The filtered extract was analyzed using a spectrophotometer (Evolution 201 UV-Visible Spectrophotometers, Thermo Scientific, Waltham, MA, USA) at wavelengths of 664 and 649 nm. The following equations were used for quantification: Chl *a* = 13.36 (A664)-5.19 (A649); Chl *b* = 27.43(A649)-8.12(A664); Chl total=17.32(A649)+7.18(A664) (Dumbravă et al., 2012). The results were expressed as µg of fresh weight unit.

#### 2.2.2 Physiological measurements

Leas gas exchange parameters included net photosynthetic rate (A^n^), intercellular CO_2_ concentration (C_i_), stomatal conductance (g_s_), and transpiration rate (E). Chlorophyll fluorescence parameters included the effective quantum yield of photosystem II (ΦPSII), photochemical quenching (qP), and the maximum efficiency of PSII photochemistry in the light (Fv′/Fm′).

All measurements were performed in the morning (from 10 a.m. to 12 a.m.) using an open gas exchange system equipped with a leaf chamber fluorometer and LED light source (LI-6400XTF, LI-COR Inc., Lincoln, NE, USA) (detection area 2 cm^2^).

Measurements were carried out under standardized saturating light conditions: PAR at 1,000 µmol m^-^² s^-^¹, leaf temperature maintained at 20 °C, ambient CO_2_ concentration set at 400 µmol mol^-^¹, and an air flow rate of 300 cm³ s^-^¹.

The steady-state fluorescence (Fs) was first established by applying an actinic light source (625 µmol m^-2^ s^-1^) until the signal reached a stable level. Next, the light-adapted maximum fluorescence (Fm′) was measured by applying a saturating pulse (>7000 µmol m^-2^ s^-1^) of light, which ensured the photosystem was light-saturated and all primary acceptors (*Q_A_*) were reduced (‘closed’). Finally, the minimal fluorescence of a light-adapted leaf (Fo′) was measured when the actinic light was briefly turned off in the presence of far-red light (“dark pulse”). The far-red light selectively oxidizes (re-opens) the *Q_A_* pool, establishing the true Fo′ level for the light-adapted state.

Fv’ (variable fluorescence) was the difference between Fo’ and Fm’. The ΦPSII and Fv’/Fm’ parameters were calculated as (Fm’-Fs)/Fm’ and (Fm’-Fo’)/Fm’; the photochemical quenching coefficient (qP) was calculated as (Fm’-Fs)/(Fm’-Fo’).

#### 2.2.3 Folate and nitrate concentration, antioxidant compounds and capacity

Folate, nitrate, antioxidant (phenol, flavonoid, carotenoid, anthocyanin, phytosterol) concentrations, and antioxidant activity were determined on lyophilized samples (ScanVac, Chemie s.r.l., Valenzano, Bari, Italy), ground using a mill (IKA, Labortechnik, Staufen, Germany). Freeze-drying chamber conditions of freeze-drying process: vacuum less than 0.2 mbar; condenser temperature -54 °C.

##### 2.2.3.1 Folate concentration

Folate were identified and quantified as reported by Czarnowska-Kujawska et al. (2022) with some modifications. Freeze-dried sample (0.3 g) was inserted into a 50 mL amber tube with 20 mL of extraction buffer (0.1 M phosphate buffer at pH 6.1), containing 0.1% (v/v) 2-mercapto-ethanol and 1% (w/v) ascorbic acid. The sample was mixed for 1 min, then boiled for 15 min, cooled in ice immediately, and centrifuged at 6,700 rpm for 15 min. The sample was then transferred into a water bath at 100 °C (15 min) and shaken three times. Finally, the sample was cooled at room temperature (*ca.*10 min).

To digest the carbohydrates and the de-conjugating folates, 1 mL of α-amylase solution (20 mg mL^-1^) and 0.25 mL of rat plasma conjugase were added to each sample, which was afterwards incubated for 4 h at 37 °C (Patring et al., 2005). Fresh rat plasma (Sigma Aldrich, St. Louis, MO, USA), used as folate conjugase source, was previously dialyzed under stirring in 0.05 M phosphate buffer, pH 6.1, with 0.1% (v/v) 2-mercaptoethanol for 12 h at 4 °C to remove endogenous folate, and stored in aliquots (1 mL) at -20 °C.

After incubation, each sample was heated in a water bath at 100 °C for 5 min to inactivate enzymes. The sample was then cooled in ice for 10 min and subsequently centrifuged at 4 °C and 6,700 rpm for 30 min. Supernatants were transferred to 50 mL amber tubes, the pellet was re-dissolved in 20 mL of extraction buffer, and re-centrifuged (6,700 rpm for 30 min at 4 °C). Supernatants were then added to the previous amber tubes. Samples were stored at -80 °C until the purification step by Solid Phase Extraction (SPE) with Strata SAX 55µm, 70A, 500 mg-6 mL tubes (Phenomenex, Castel Maggiore, BO, Italy). The cartridges were conditioned with 5 mL of methanol and 5 mL of water. Subsequently, extract volumes were applied with a flow rate of 1 drop sec^-1^.

To remove the matrix-interfering components, the cartridges were rinsed with 5 mL of water, and the retained folates were eluted with sodium acetate (0.1 M), containing 10% (w/v) sodium chloride and 0.1% (v/v) 2-mercaptoethanol. Samples were collected for HPLC analysis.

Chromatographic analyses were performed by an HPLC system (Agilent-1100 Series, Palo Alto, CA, USA) consisting of a quaternary pump for gradient elution equipped with a micro-vacuum degasser, an auto-sampler connected to an injection valve with a loop of 100 μL, a thermostated column compartment, a Luna C18(2), 100 Å column (4.6×150 mm, 5 μm i.d.) analytical column with a security guard cartridge (Phenomenex, Torrance, California, USA), a diode array detector, and a fluorescence detector. Signals were recorded by a ChemStation computer software (Agilent, Palo Alto, CA, USA). HPLC analyses were achieved by a binary gradient elution at 1 mL min^-1^ using a mobile phase constituted by methanol (eluent A) and 0.1% formic acid in water (eluent B). The gradient elution program was as follows: initially, a ratio of 5:95 v/v (A:B) was maintained for the first 0-8.0 min, followed by a transition to a ratio of 18:82 v/v (A:B) through the subsequent 8.0-17.0 minutes.

This ratio was then maintained until 36 min, followed by the late step of ratio 5:95 v/v (A:B), maintained until the end of the run. The eluate was monitored by both a fluorescence detector set at an excitation wavelength of 290 nm and an emission wavelength of 360 nm, and a diode array detector set at 290 nm.

Spectral analyses of folic acid (PteGlu), tetra-hydro-folate (H4-folate), 5-methyl-tetra-hydro-folate (5-CH3-H4-folate), and 5-formyl-tetra-hydro-folate (5-HCO-H4-folate) standards (Schircks Laboratories, Jona SG, Switzerland) and real samples were performed to verify the method selectivity. Standard solutions were previously stored at -20 °C, and the calibration solutions were prepared before use by dilution of the stock solution with water and 0.1% formic acid.

Values were determined from a calibration curve (0-10 mg L^-1^; R^2^ = 0.998) using standard solutions, previously stored at -20 °C; the calibration solutions were prepared before use by dilution of the stock solution with water and 0.1% formic acid. The results were expressed as µg of folates per dry weight unit.

##### 2.2.3.2 Nitrate concentration

The concentration of NO_3_-N was determined as reported by Bonasia et al. (2024) by ion chromatography (Dionex ICS 3000; Dionex ThermoFisher Scientific, Waltham, MA, USA) after extraction from sample (0.5 g) with 50 mL of eluent solution in a shaking water bath at room temperature for 30 min. The mixture was filtered twice through no. 2 Whatman paper and then through a 0.22 μM Millipore filter, before injection into the ion chromatography system. The eluent consisted of 3.5 mM sodium carbonate and 1 mM sodium bicarbonate (flow rate, 1 mL min^-1^).

The system was equipped with an isocratic pump, a model AS-DV autosampler, a self-generating ASR anion suppressor (4 mm), A Dionex Ion-Pac AS23 (Dionex Corp., Milan, Italy) analytical column (4 mm×250 mm), and a guard column (4 mm×50 mm), maintained at 35 °C. Values were determined from a calibration curve (1-100 mg L^-1^; R2 = 0.998) prepared with dilution of anion standard solutions (ThermoFisher Scientific, Waltham, MA, USA). The results were expressed as mg of NO_3_ per fresh weight unit.

##### 2.2.3.3 Antioxidant compounds

###### 2.2.3.3.1 Phenols

Total phenols were extracted from sample material (30 mg) using 1 mL of water/methanol (20:80 v/v) at room temperature in an ultrasonic cleaner bath (DU-32 Digital; Argo Lab, Carpi, MO, Italy) for 15 min. The mixture was then centrifuged in a refrigerated centrifuge (ThermoFisher Scientific, Waltham, MA, USA) at 14,000 rpm for 15 min at 4 °C, and the supernatant was collected. This extraction process was repeated twice, and the combined supernatants were stored at -20 °C for analysis within 24 hours.

Phenolic concentration was determined spectrophotometrically on methanolic extracts using the Folin-Ciocalteu method (Singleton and Rossi, 1965): 100 mL of the extracts was diluted with 3 mL of distilled water, mixed with 0.5 mL of Folin-Ciocalteu reagent, and kept at room temperature for five min; then 1.0 mL of 20% Na_2_CO_3_ was added to the mixture. After 45 min at 30 °C, absorbance was read at 750 nm (Shimadzu UV-1800, Shimadzu Scientific Instruments, North America, USA). Values were determined from a calibration curve (0-250 mg L^-1^; R^2^ = 0.998) prepared with gallic acid standard solutions using the same procedure as for the extracted sample. The results were expressed as mg of acid gallic equivalent per fresh weight unit.

###### 2.2.3.3.2 Anthocyanins

For total anthocyanin determination, the same extract solution was used, following the method reported by Sims and Gamon (2002). Absorbance was measured at 650 nm and 525 nm using the spectrophotometer. To correct for chlorophyll interference, the following equation was used: AA=A529-(0.288*A650), where AA represents the corrected anthocyanin absorbance. Total anthocyanin content was expressed as cyanidin-3-glucoside equivalent, using the corrected absorbance and a molar absorbance coefficient for anthocyanin at 525 nm of 26.900 L mol^-1^ cm^-1^ (Murray and Hackett, 1991). The results were expressed as mg of cyanidin-3-glucoside equivalent per fresh weight unit.

###### 2.2.3.3.3 Flavonoids

Flavonoid content was determined using the AlCl3 method (Chandra et al., 2014). Total flavonoid extraction was carried out on sample material (30 mg) in water/methanol (1 mL) (20:80 v/v) at room temperature in an ultrasonic cleaner bath (DU-32 Digital; Argo Lab, Carpi, MO, Italy) for 15 minutes. The mixture was then centrifuged in a refrigerated centrifuge (ThermoFisher Scientific, Waltham, MA, USA) at 14,000 rpm for 15 min at 4 °C, and the supernatant was collected. The extraction was repeated twice, and the combined supernatants were stored at -20 °C for analysis within 24 hours. In detail, 200 µL of the extract or diluted standard quercetin solutions (1-100 mg L^-1^) were mixed with 40 µL of 10% AlCl3, 40 µL of 1 M potassium acetate, 1,120 µL of distilled water. Samples were incubated for 30 minutes at room temperature. The absorbance was measured on UV-Vis spectrophotometry at a wavelength of 415 nm. The results were expressed as mg of quercetin equivalent per fresh weight unit.

###### 2.2.3.3.4 Vitamin C

Vitamin C content - L-ascorbic acid (AA) + L-dehydroascorbic acid (DHAA) - was extracted by homogenizing 0.1 g of sample material for 15 min with 5 mL of methanol/water (5:95 v/v), plus citric acid (21 g L^-1^), EDTA (0.5 g L^-1^) and NaF (0.168 g L^-1^). The homogenate was filtered through two layers of cheesecloth and the pH was adjusted to 2.2-2.4 by the addition of HCl (6 mol L^−1^). After centrifugation (12,000 g, 5 min, 4 °C), the supernatant was filtered through a Sep-Pak C18-cartridge (Waters, Milford, MA, USA) and then through a 0.2 μm cellulose acetate filter (INCOFAR, Modena, Italy). AA and DHAA contents were determined as described by Zapata and Dufour (1992) with some modifications.

The HPLC analysis was achieved after the derivatization of DHAA into the fluorophore 3-(1,2-dihydroxyethyl)furol[3,4-b]quinoxaline-1-one (DFQ), with 1,2-phenylenediamine-dihydrochloride (OPDA). The extracts (20 μL) were analyzed with an HPLC (Agilent Technologies 1200 Series; Agilent, Waldbronn, Germany) equipped with a DAD detector and a binary pump. The separations of DFQ and AA were achieved on a Synergi 4u-Hydro-RP 80A C18-column (250x4.60 mm) (Phenomenex Inc., Castel Maggiore, BO). The mobile phase was a methanol:water solution (5:95 v/v), containing 5 mmol L^-1^ of cetrimide and 50 mmol L^-1^ KH_2_PO_4_ at pH 4.5. The flow rate was 1 mL s^-1^. The detector wavelengths were 348 nm for DHAA and 251 nm for AA. The total vitamin C is the sum of AA and DHAA. The results were expressed as mg of vitamin C per fresh weight unit.

###### 2.2.3.3.5 Carotenoids

Total carotenoids were extracted as reported by Taungbodhitham et al. (1998), as follows: MgCO_3_ (0.05 g) was added to 0.1 g of the sample to neutralize cytosolic acids; 0.01 g of celite was used for better tissue disruption. The extraction was with 10 mL of ethanol:hexane (4:3 by volume); 1 mL of pyrogallol solution (5%) was added as an antioxidant. The mixture was placed in a mechanical shaker for 15 min and then centrifuged at 6,700 rpm for 10 min, and the supernatant was collected. The residue was re-extracted; the two extracts were combined and decanted into a 50 mL tube. The supernatant hexane phase was transferred into another tube, and the lower aqueous phase was discarded. To overcome the problem of carotenoid overestimation by the presence of chlorophylls, a saponification step was included during extraction. In brief, an equal volume of 10% methanolic KOH was added to the recovered hexane phase, the mixture was shaken vigorously for 1 min, and then placed on ice for 15 min. After centrifugation at 6,700 rpm for 10 min, the supernatant (hexane phase) was collected and washed two times with 15 mL of NaCl 10% solution and two times with 15 mL of water. The aqueous phase was discarded, and the extracts were measured at 450 nm using a UV-visible spectrophotometer and estimated according to the ß-carotene calibration curve (Biehler et al., 2010).

The results were expressed as mg of ß-carotene equivalent per fresh weight unit.

###### 2.2.3.3.6 Phytosterols

Total phytosterols were quantified as reported by Xiang et al. (2016) with some modifications. Briefly, 100 mg of sample material was weighed into a screw-cap tube, then 3 mL of sulfuric acid solution (0.5 mol L^-1^ in 95% ethanol) was added, and the mixture was vortexed for 10 seconds. The thermal decomposition of phytosterols was prevented by adding tert-butyl-hydro-quinone (3 mg). Acid hydrolysis was performed at 55 °C for 1 h. Alkali saponification was carried out by adding 3 mL of sodium hydroxide solution (2.0 mol L^-1^ in 95% ethanol); alkali saponification occurred at 55 °C for 1 h. Then, 3 mL of sodium chloride solution (1.0 mol L^-1^) was added to prevent emulsification and facilitate hexane extraction.

A double extraction was performed by adding hexane (5 mL). The two supernatants were combined after stirring the mixture (15 minutes). The extract was then dried using a rotary evaporator (Strike 300, Steroglass, San Martino in Campania, AV, Italy) and subsequently dissolved in of absolute ethanol (1.0 mL) to create the crude extract, which was purified by solid phase extraction (SPE) column with a 500 mg-C18-cartridge (Strata C18-E, Phenomenex, Castel Maggiore, BO, Italy). The complete SPE procedure includes methanol activation, water equilibrium, sample loading and elution.

The concentration of total phytosterols was measured according to the spectrophotometry method described by Feng et al. (2020): 10 g of FeCl_3_ 6H_2_O was placed into a 100 mL volumetric flask and diluted with phosphoric acid (85%) to reach the volume. Next, 1.5 mL of the FeCl_3_ solution (10%) was transferred to a 100 mL volumetric flask and diluted with concentrated sulfuric acid. Subsequently, the present sulfuric-phosphoric-FeCl_3_ reagent (2 mL) was combined with stigmasterol in ethanol solutions (4 mL) at a concentration ranging from 19 to 45.5 μg mL^-1^ and with the sample extract solution (4 mL).

The reaction proceeded for 15 minutes; afterwards, the absorption was measured using a spectrophotometer (Evolution 201 UV-Visible Spectrophotometers, Thermo Scientific, Waltham, MA, USA) at a wavelength of 520 nm. The concentration was calculated based on a standard curve. The results were expressed as μg of stigmasterol equivalent per unit fresh weight.

#### 2.2.3.4 Antioxidant capacity

Radical scavenging capacity by the TEAC(ABTS) method was assessed using a slightly modified version of the TEAC (Trolox Equivalent Antioxidant Capacity) method described by Re et al. (1999), including the de-colorization assay applied for both hydrophilic and lipophilic antioxidants. This method is based on the capacity of antioxidants to neutralize the radical cation blue/green ABTS^+•^ chromophore to a colorless ABTS form. The ABTS^+•^ form is prepared by an oxidation reaction of ABTS with potassium persulfate. A decrease in the concentration is linearly dependent on the antioxidant concentration.

The lyophilized sample (30 mg) was extracted with 1 mL of 80% methanol in DU-32 Digital ultrasonic power (Argo Lab, Carpi, MO, Italy) for 15 min. The mixture was centrifuged at 13,000 rpm for 10 min, and the supernatant was collected. The extraction was repeated twice, and the two supernatants were combined into a hydrophilic fraction. Subsequently, the residue was extracted twice with 1 mL of hexane in ultrasonic power for 15 min and the two supernatants were combined into the lipophilic fraction. The extracts were stored at 20 °C and measured within 24 h. The absorbance was measured at 734 nm after 6 min standing.

Values were determined from a calibration curve prepared with Trolox (6-hydroxy-2,5,7,8-tetramethylchroman-2-carboxylic acid) solutions (0-15 µM of Trolox) prepared following the same procedure as for the extracted sample. The results were expressed as µmol of Trolox equivalent per unit fresh weight.

### 2.3 Statistical analysis

Statistical analysis was conducted using the General Linear Model procedure of the SAS software (SAS 9.1; SAS Institute, Cary, NC, USA). Mean comparisons were performed using the least significant difference (LSD) test at a significance level of p=0.05.

## 3 Results

### 3.1 Bio-physical measurements

The application of foliar elicitors (salicylic acid-SA and phenylalanine-PA) did not affect key production parameters. Across all treatments, average fresh yield remained consistent at 1.0 kg m^-2^, with mean leaf area measuring 30.2 cm^2^ and an average of 4.0 leaves per plant (**Table 1**). No significant treatment effects on leaf appearance parameters were detected through instrumental color analysis. The average values across all treatments were 60.2 for lightness (L*), 136.5 for hue angle (h°), and 24.4 for chroma (C*) (**Table 1**). The dry matter concentration (DM) of control plants was not different from elicitor-treated ones (**Table 1**), while PA-treatment showed a lower DM than SA-plants (**Table 1**). SA-treatment reduced chlorophyll (total and component *a* and *b*) concentration in comparison to control (-20, -15, -28%) along with SPAD index (-4%) (**Table 1**).

**Table 1:** Effect of folate elicitors (salicylic acid, SA; phenylalanine, PA) on physical and bio-physiological traits of kale (‘Cavolo Nero’) baby leaves.

Elicitor	FY^3^ (kg m^-2^)	DM^3^ (g kg^-1^ f.w.)	LA^3^ (cm^2^)	LN^3^ (n.)	L*	h°	C*	SPAD	CHL *a*	CHL *b*	CHL total
					(-)^3^				(µg g^-1^ f.w.)^3^		
Control	1.12 ± 0.17a^2^	84.6 ± 3.1ab	32.0 ± 4.4a	4.0 ± 0.06a	60.9 ± 0.4a	131.4 ± 7.2a	25.1 ± 0.9a	49.5 ± 0.85a	72.7 ± 10.4a	57.2 ± 8.1a	126.7 ± 18.5a
SA	0.90 ± 0.14a	86.6 ± 2.0a	27.4 ± 5.4a	3.9 ± 0.03a	60.2 ± 0.8a	133.2 ± 6.4a	24.4 ± 1.8a	47.7 ± 0.61b	58.0 ± 3.4b	41.1 ± 5.0b	94.4 ± 6.2b
PA	1.10 ± 0.17a	82.2 ± 1.9b	31.2 ± 8.2a	4.2 ± 0.32a	59.4 ± 0.7a	145.0 ± 7.2a	23.7 ± 0.8a	50.3 ± 1.25a	64.0 ± 5.1ab	50.7 ± 5.9ab	108.6 ± 10.0ab
Significance^1^	n.s.	*	n.s.	n.s.	n.s.	n.s.	n.s.	*	*	*	*

^1^n.s.,*, not significant or significant at p ≤ 0.05.

^2^Means (± SE) in columns not sharing the same letters are significantly different according to the LSD test (p=0.05).

^3^ FY, fresh yield; DM, dry matter concentration; LA, leaf area; LN, leaf number; L*, lightness index; h°, hue angle; C*, chroma index; SPAD, Soil Plant Analysis Development index; CHL, chlorophyll. Values are the mean of eighteen subsamples (n = 18).

### 3.2 Physiological measurements

The photosynthetic rate (An; 13.9 mol m^-2^ s^-1^, on average) and the chlorophyll *a* fluorescence parameters - maximum quantum yield of PSII (Fv’/Fm’; 0.59, on average); the efficiency of PSII (ΦPSII; 0.17, on average), and the photo-chemical quenching (qP; 0.28, on average) were not affected by elicitor treatments (**Table 2**). Stomatal conductance (gs) and transpiration rate (E) of control plants were not different from elicitor-treated ones (**Table 2**), while PA-treatment showed a lower gs and E than SA-plants (**Table 2**). The intracellular CO_2_ concentration (Ci) of PA-plants was lower than that of control and SA-treated plants (**Table 2**).

**Table 2:** Effect of folate elicitors (salicylic acid, SA; phenylalanine, PA) on photosynthetic gas exchange and chlorophyll fluorescence of kale (‘Cavolo Nero’) baby leaves.

Elicitor	Net assimilation rate (An) (μmol CO_2_ m^-2^ s^-1^)	Stomatal conductance to H_2_O (gs) (mol H_2_O m^-2^ s^-1^)	Inter- cellular [CO_2_] concentration (Ci) (μmol CO_2_ mol^-1^)	Transpiration rate (E) (mmol H_2_O m^-2^ s^-1^)	Maximum quantum yield of PSII (Fv’/Fm’) (-)	Efficiency of PSII (ΦPSII) (-)	Photo-chemical quencing (qP) (-)
Control	13.9 ± 1.16a^2^	0.66 ± 0.05ab	347 ± 2.96a	6.7 ± 0.27ab	0.60 ± 0.02a	0.16 ± 0.01a	0.27 ± 0.03a
SA	14.0 ± 0.71a	0.76 ± 0.05a	350 ± 2.88a	7.2 ± 0.25a	0.58 ± 0.02a	0.17 ± 0.01a	0.30 ± 0.02a
PA	13.7 ± 0.49a	0.58 ± 0.09b	338 ± 3.48b	6.3 ± 0.60b	0.60 ± 0.02a	0.17 ± 0.01a	0.28 ± 0.02a
Significance^1^	n.s.	*	*	*	n.s.	n.s.	n.s.

^1^n.s.,*, not significant or significant at p≤ 0.05.

^2^Means (± SE) in columns not sharing the same letters are significantly different according to the LSD test (p=0.05). Values are the mean of twelve subsamples (n = 12).

### 3.3 Folate, anti-nutritional, and antioxidant compounds and capacity

#### 3.3.1 Folate and nitrate concentration

PA- and SA-treated leaves had 7% higher total folate content (1,283 µg 100 g^-1^ d.w., on average) as the sum of 5-formyl-tetrahydrofolate [5-f-THF] and 5-methyl-tetrahydrofolate [5-m-THF] compared to the untreated plants (1,203 µg 100 g^-1^ d.w.) (**Table 3**). SA-treated plants showed a higher 5-m-THF content than control ones (**Table 3**). The 5-f-THF levels were not affected by treatments, averaging 551 µg 100 g^-1^ d.w. (**Table 3**). The folic acid (PteGlu) and the tetra-hydro-folate (H4-folate) were not detected (**Table 3**). PA-treated plants accumulated higher nitrate than the control plants and SA-treated plants (+56%) (**Table 3**).

**Table 3:** Effect of folate elicitors (salicylic acid, SA; phenylalanine, PA) on folate and nitrate concentration of kale (‘Cavolo Nero’) baby leaves.

Elicitor	Folates			Nitrate (mg kg^-1^ f.w.)
	5-methyl-tetra-hydro-folate (5-m-THF) (5-CH_3_-H_4_-folate)	5-formyl-tetra-hydro-folate (5-f-THF) (5-HCO-H_4_-folate)	Total	
	(µg 100 g^-1^ d.w.)			
Control	665 ± 32.2b^2^	538 ± 17.4a	1,203 ± 22.2b	2,555 ± 203b^2^
SA	738 ± 13.2a	538 ± 12.0a	1,277 ± 13.1a	2,519 ± 145b
PA	712 ± 12.3ab	576 ± 29.4a	1.289 ± 27.0a	3,954 ± 239a
Significance^1^	*	n.s.	*	***

^1^n.s.,*, and ***, not significant or significant at p ≤ 0.05 and 0.001.

^2^Means (± SE) in columns not sharing the same letters are significantly different according to the LSD test (p=0.05). Values are the mean of twelve subsamples (n = 12).

#### 3.3.2 Antioxidant compounds and capacity

SA-treatment decreased phytosterol and anthocyanin (ANT) concentrations, while increasing the level of carotenoids (CAR) and vitamin C compared to control and PA-leaves (**Table 4**).

**Table 4:** Effect of folate elicitors (salicylic acid, SA; phenylalanine, PA) on antioxidant status of kale (‘Cavolo Nero’) baby leaves.

Elicitor	Phytosterols (mg s.e. kg^-1^ f.w.)^3^	TP^2^ (mg a.g.e. 100 g^-1^ f.w.)^3^	FLAV^2^ (mg q.e. 100 g^-1^ f.w.)^3^	ANT^2^ (mg c.g.e. 100 g^-1^ f.w.)^3^	CAR^2^ (mg 100 g^-1^ ßc.e. f.w.)^3^	VIT-C^2^ (mg kg^-1^ f.w.)	Antioxidant capacity^2^		
							HAC^2^	LAC^2^	TAC
							(µmol T.E. g^-1^ f.w.)^3^		
Control	24.3 ± 1.3a^4^	77.9 ± 2.5a	28.2 ± 2.1ab	4.7 ± 0.65a	3.2 ± 0.25b	60.5 ± 11.2b	7.1 ± 0.37a^2^	1.1 ± 0.07a	8.2 ± 0.40a
SA	21.4 ± 0.7b	75.0 ± 1.3a	30.9 ± 3.9a	3.4 ± 0.40b	3.8 ± 0.25a	114.2 ± 9.0a	6.7 ± 0.50ab	1.2 ± 0.05a	7.9 ± 0.52ab
PA	24.5 ± 1.1a	71.9 ± 1.3b	22.7 ± 3.0b	4.6 ± 0.67a	3.3 ± 0.22b	30.3 ± 6.3c	6.4 ± 0.18b	1.1 ± 0.08a	7.5 ± 0.18b
Significance^1^	*	*	*	*	*	***	*	n.s.	*

^1^n.s., * and *** not significant or significant at p ≤ 0.05 and 0.01.

^2^TP, total phenolic content; FLAV, total flavonoid content; CAR, total carotenoid content; ANT, total anthocyanin content; VIT-C, vitamin C; TAC, HAC and LAC, total, hydrophilic and lipophilic antioxidant capacity.

^3^s.e, stigmasterol equivalent; a.g.e, acid gallic equivalent; q.e, quercetin equivalent; ßc.e., ß-carotene equivalent; c.g.e, cyanidin-3-glucoside equivalent; T.E., trolox equivalent.

^4^Means (± SE) in columns not sharing the same letters are significantly different according to the LSD test (p=0.05). Values are the mean of twelve subsamples (n = 12).

In PA-treated leaves, flavonoids (FLAV) were lower than those of control plants, and they showed the lowest level of phenols (TP) and vitamin C in comparison to control and SA treatment (**Table 4**).

Regarding antioxidant capacity, PA-treated leaves showed lower total (TAC) and hydrophilic (HAC) activity compared to the control (**Table 4**), while the lipophilic component (LAC) remained unaffected across treatments (1.1 µmol T.E. g^-1^ f.w., on average) (**Table 4**).

## 4 Discussion

### 4.1 Folates in kale baby leaves

The composition of folates in plants includes di-hydro-folates, and several tetra-hydro-folates (THF), among which 5-methyl-H4-folate (5-m-THF) and 5-formyl-H4-folate (5-f-THF) are the most representative, followed by 5-formimino-H4-folate, 10-formyl-H4-folate, 10-formimino-H4-folate, 5,10-methylene-H4-folate, 5,10-methenyl-H4-folate (Kołton et al., 2022). Our HPLC-based folate profiling (**Table 3**) aligns with previous findings - obtained with the same analytical method- in Brassicaceae, particularly cauliflower (Czarnowska-Kujawska et al., 2022). This consistency underscores the critical influence of genotype, a trend also observed in other crops such as maize (Paul et al., 2025) and legumes (Jha et al., 2015).

Regardless of the specific elicitation treatment, our kale baby leaves, hydroponically-grown under greenhouse autumn Mediterranean conditions, showed a higher level of folate (110 μg 100 g^-1^, on fresh weight basis) than the mature counterpart *Brassica oleracea* (Acephala Group) (62 μg 100 g^-1^ f.w.) and broccoli (*Brassica oleracea* var. *italica* L.) (63 μg 100 g^-1^ f.w.), according to the National Nutrient Food Database for Standard Reference Release 28 (SR28 released in April 2018) (USDA, 2018).

Kale baby leaves can be classified at ‘medium’ folate level when compared to other vegetable species, which span from ‘very low’ (e.g., raw potato, 15 μg 100 g^-1^ f.w.), to ‘high’ (spinach, 194 μg 100 g^-1^ f.w.) or ‘very high’ (raw lentils, 479 μg 100 g^-1^ f.w) (Strobbe and Van Der Straeten, 2017). When compared to experimental data from Johansson et al. (2007) on folate concentration of leafy greens, kale baby leaves exhibited higher folate levels than lettuce (averaging 69.1 μg 100 g^-1^ f.w. across 7 morphotypes), but lower than other leafy species such as spinach (174 μg 100 g^-1^ f.w.) and rocket (162 μg 100 g^-1^ f.w.), the latter being a member of the same family of kale (Brassicaceae).

The Recommended Dietary Allowances (RDA) for a day for folate are 400 μg for adults, and 300 μg for children, while it is raised to 600 μg for pregnant women and 500 μg for breastfeeding women, being included in the segment of the population at risk for folate deficiency (FAO/WHO, 2002).

When considering folate-fortified kale baby leaves consumed raw (experiencing no preparation losses), a single serving of 200 g would fulfil 52, 69, 34, 41% of the daily folate requirement, respectively, for adults, children, pregnant women and breastfeeding women.

### 4.2 Effect of SA and PA elicitors on folate fortification in kale baby leaves

Under the experimental conditions of this study, foliar elicitation with salicylic acid (SA, 250 µM) and phenylalanine (PA, 0.37 µM) led to a significant, but moderate increase in total folate content in 37-day-old kale baby leaves. Previous studies have reported more substantial folate enhancement in other leafy greens.

In mature coriander, foliar SA application (250 µM) doubled total folate levels, increasing concentrations from 1,330 to 2,867 µg 100 g^-1^ d.w (Puthusseri et al., 2013). The primary differences between our findings and existing literature may stem from varying genotype responses and the earlier developmental stage of baby leaves. In the study by Puthusseri et al. (2013). the use of 50-day-old coriander leaves likely provided a larger absorbing surface area, potentially leading to a more efficient foliar application. Furthermore, the higher temperature and light regimes in their study may have enhanced biofortification efficiency; indeed, established evidence confirms that light conditions significantly influence folate biosynthesis in plants (Rébeillé et al., 2006; Gorelova et al., 2017).

Regarding PA, there is currently no literature confirming its efficacy as a folate elicitor when applied via foliar spray. In the only relevant study, Watanabe et al. (2017) provided 2 mM of PA through the nutrient solution of hydroponically grown spinach (25-day-old leaves). This root-based delivery likely facilitated continuous absorption, leading to a two-fold increase in folate content from 150 to 306 µg 100 g^-^¹ f.w. In contrast, the foliar application used in our study - coupled with a limited absorbing leaf area (30 cm^2^, **Table 1**) - may have resulted in less effective elicitation. While Teixeira et al. (2018) previously studied foliar PA application in greenhouse soybean, their research did not focus on folate biofortification, making a direct comparison impossible.

The comparatively modest folate enhancement observed in our study suggests the existence of species-specific differences in the regulation of folate biosynthesis. Moreover, the complex interactions between genotype, elicitor parameters (type, dosage, and application method), and growing conditions should not be overlooked, as these variables significantly influence the mechanistic pathways underlying elicitor-induced folate accumulation.

Our evidence suggests that light may be a critical factor; specifically, under Mediterranean conditions, spring cultivation cycles could yield superior results due to more favorable natural light dynamics for both plant growth and elicitor activity. Furthermore, folate enhancement could be amplified through light modulation -specifically by utilizing the red-light spectrum (Chang et al., 2021; Xie et al., 2022) - and/or supplementing light intensity, as supported by recent studies (Okazaki and Yamashita, 2019).

The primary objective of this work was not to investigate dose-response relationships, but rather to evaluate the efficacy of two different classes of elicitors. However, optimizing application dosages warrants further attention to maximize the success of folate biofortification.

Despite PA and SA exhibiting a similar elicitation effect, kale treated with foliar applications of SA (250 µM) showed a more pronounced increase in 5-m-THF content (**Table 3**). This finding is nutritionally relevant because 5-m-THF represents the bioactive folate form readily utilized by the human body (Verhaar et al., 1998). Although folic acid (PteGlu), tetra-hydro-folate (H4-folate) are commonly reported in leafy vegetables, they were not detected in our kale extracts. Their absence may be attributed to their high instability or to the specific growth conditions and baby leaf stage of the plants (**Table 3**).

While the absolute increase in folate content may appear moderate, the main nutritional advantage lies in the preservation of folate integrity. Unlike conventional leafy vegetables, which often undergo significant thermal degradation during cooking, these biofortified baby leaves maintain their full nutrient potential as they are usually consumed raw. This makes them particularly valuable for populations with elevated requirements, such as pregnant women. Consequently, the synergy between increased 5-m-THF content levels and the avoidance of cooking losses establishes these leaves as a potentially highly effective dietary folate source.

### 4.3 Effect of SA and PA elicitors on bio-physical and physiological traits of kale baby leaves

The elicitors applied in our study effectively enhanced total folate concentration (**Table 3**), while maintaining both yield (**Table 1**) and visual quality. Both elicitors preserved the instrumental key parameters of color (**Table 1**), which is an important external attribute influencing consumer purchase decisions (Rajain and Rathee, 2019). Although the SA application induced a reduction in chlorophyll content on a fresh weight basis, the increase in dry matter concentration (DM) resulted in a concentration effect of the pigments. While this effect was not captured by instrumental measurements, it allowed the leaves to maintain a stable green color (**Table 1**). Moreover, DM represents a critical quality attribute, as it significantly influences leaf consistency - a strategic factor for the shelf-life and processing of baby leaf vegetables.

PA treatment promoted stomatal closure (reduced gs values), with a parallel reduction in transpiration and intracellular CO_2_ concentration. However, PA-treated plants maintained comparable net CO_2_ assimilation rates (An) in relation to the other treatments (**Table 2**). On the contrary, SA did not alter any gas exchange parameters (**Table 2**).

The ΦPSII parameter represents the operating efficiency of PSII in the light, indicating the fraction of the absorbed light actually used for photochemistry processes. It depends on its components: (a) Fv’/Fm’ (maximum PSII photochemical efficiency in the light, if all PSII centers were open), which is a sensitive indicator of how well PSII can convert light energy into chemical energy; and (b) qP (photo-chemical quenching coefficient), measuring the fraction of PSII centers that are open. Together, these parameters provide a comprehensive picture of PSII function.

Since the indices of chlorophyll *a* fluorescence parameters in light-adaptation conditions were not affected by elicitor application as well as An (**Table 2**), it is possible to argue that photosynthetic efficiency remained stable across all treatments.

### 4.4 Effect of SA and PA on anti-nutritional and antioxidant profile, and capacity of kale baby leaves

#### 4.4.1 Nitrate

Although dietary nitrate-abundant in baby leaves like spinach and rocket salad offers potential metabolic benefits such as lowered blood pressure (Jonvik et al., 2016) and ergogenic effects (Macuh and Knap, 2021), its consumption is not without risk. Due to these health concerns, regulatory limits have been imposed. The European Commission’s Regulation (EU 915, 2023) sets maximum nitrate levels for leafy vegetables to mitigate the risks of excessive intake (some examples: spinach, 3,500 mg kg^-1^ f.w.; autumn greenhouse lettuce, 5,000 mg kg^-1^ f.w.; autumn rocket, 7,000 mg kg^-1^ f.w.).

Our research showed significant variation in nitrate accumulation depending on elicitor type. The SA application maintained nitrate levels compared to the control plants, while the PA foliar application increased nitrate content in kale leaves. PA is reported to influence nitrate reductase (NR) activity (the key enzyme catalyzing the first step of nitrate assimilation) in a multi-faceted manner, through different mechanisms as well as by feedback inhibition of enzymes involved in its biosynthesis (Amaya-Farfan and Pacheco, 2003), nitrogen metabolism (Jiao et al., 2018), nitrogen transport (Nazoa et al., 2003) and/or the production of downstream metabolites (as coumarins, Deng and Lu, 2017; Abenavoli et al., 2001).

Based on the classification proposed by Santamaria (2006), our kale samples (averaging 3,010 mg kg^-1^ f.w.) fall within the ‘very high’ (2,500-5,000 mg kg^-1^ f.w.) nitrate levels. However, these levels remain substantially below the maximum nitrate levels set by EU 915 (2023) for comparable autumn Brassicaceae rocket (7,000 mg kg^-1^ f.w.).

#### 4.4.2 Antioxidant profile and capacity

Baby leafy vegetables are recognized as excellent sources of antioxidant molecules, including phenolic compounds (anthocyanins, flavonoids), vitamin C, carotenoids, and phytosterols. The total antioxidant capacity (TAC), reflecting the combined efficiency of all antioxidant compounds in scavenging free radicals, encompasses both hydrophilic (HAC) (associated with anthocyanins, flavonoids, phenols, vitamin C, and others) and more negligible lipophilic (LAC) components (associated with carotenoids, phytosterols, tocopherols and others, such as lipophilic phenols).

Our study revealed distinct effects of the elicitor treatments on the antioxidant profile of kale leaves, affecting the HAC (**Table 4**). This component predominantly contributed to TAC (85%), underscoring the relative importance of hydrophilic compounds in determining the total antioxidant potential of kale leaves. The LAC was low and appeared not influent on TAC in the examined plants. The SA application enhanced vitamin C, scarcely modifying overall HAC. This apparent paradox may be explained by the simultaneous reduction in anthocyanin among the HAC components. Exogenous SA has already been proven to increase the content of some classes of phenolic compounds (Singh, 2023) and other antioxidants, such as ascorbic acid (Kobyletska and Kavulych, 2024), most probably due to the common pathways of synthesis, but findings related to the SA effect on anthocyanins are contradictory. The SA-modulated variation of anthocyanin level depends on stressing conditions (Shi et al., 2023) and/or SA dosage (by foliar spray on grapes, Yue et al., 2023), which modified the stimulating/inhibiting effect of the activity of anthocyanin synthesis enzymes.

In the SA baby-leaf, the increase in carotenoids could be counteracted by the reduction in phytosterols, both antioxidant compounds affecting LAC (**Table 4**). The SA depressed phytosterol accumulation (**Table 3**) aligning with the results of a study carried out on spadeleaf (*Centella asiatica* L. Urban) (Loc et al., 2016). The authors demonstrated that the addition of salicylic acid in spadeleaf cell culture inhibited the expression of the CaCYS gene, representing the gene responsible for the synthesis of phytosterols (Hernández-Vázquez et al., 2009).

Differently, PA-application significantly reduced HAC, primarily through decreases in total phenols and vitamin C content (**Table 4**). Previous research showed a positive relationship between PA (pre-harvest and post-harvest) application and an improved antioxidant capacity by an increase of vitamin C and phenols (Oosalo et al., 2024; Kobyletska and Kavulych, 2024) on harvest and post-harvest plant products (fruits, vegetables). To our knowledge there are no study asserting the contrary. It could be related to several factors and interactions between them: genotype and elicitor (dosage, mode of application, application period) and/or growing conditions.

## 5 Conclusion

Although the folate biofortification was not notable, the elicitors applied by foliar spraying were both effective compounds in contributing to increasing total folate concentration, while preserving yield and visual quality parameters in baby kale leaves. Among the tested elicitors, phenylalanine proved less favorable as a foliar spray, as it slightly disrupted kale physiology, resulting in a decreased antioxidant profile, an increase of nitrate levels and compromised final quality. Conversely, salicylic acid spray application emerged as a more promising pre-harvest strategy; in addition to promoting folate accumulation and profile, it improved leaf consistency and increased beneficial antioxidant compounds, such as vitamin C, without negatively affecting the product’s overall antioxidant capacity or nitrate content.

Notwithstanding the differences identified in this study, future research into foliar phenylalanine and salicylic acid elicitation should evaluate a broader range of species and genotypes across various dosages and cultivation cycles. Regarding the post-harvest phase, it is essential to monitor shelf-life and folate stability during storage. Additionally, in-depth analyses of folate bio-accessibility and bio-availability are necessary to ensure that nutritional enhancement effectively translates into tangible health benefits for consumers.

## Data Availability

The raw data supporting the conclusions of this article will be made available by the authors, without undue reservation.
