# Supplementary figures and images for: Folate biofortification strategy by elicitation in kale baby leaves
Source: Front Plant Sci. 2026 Apr 7;17:1737335. doi: 10.3389/fpls.2026.1737335 (PMC13096699; doi:10.3389/fpls.2026.1737335)

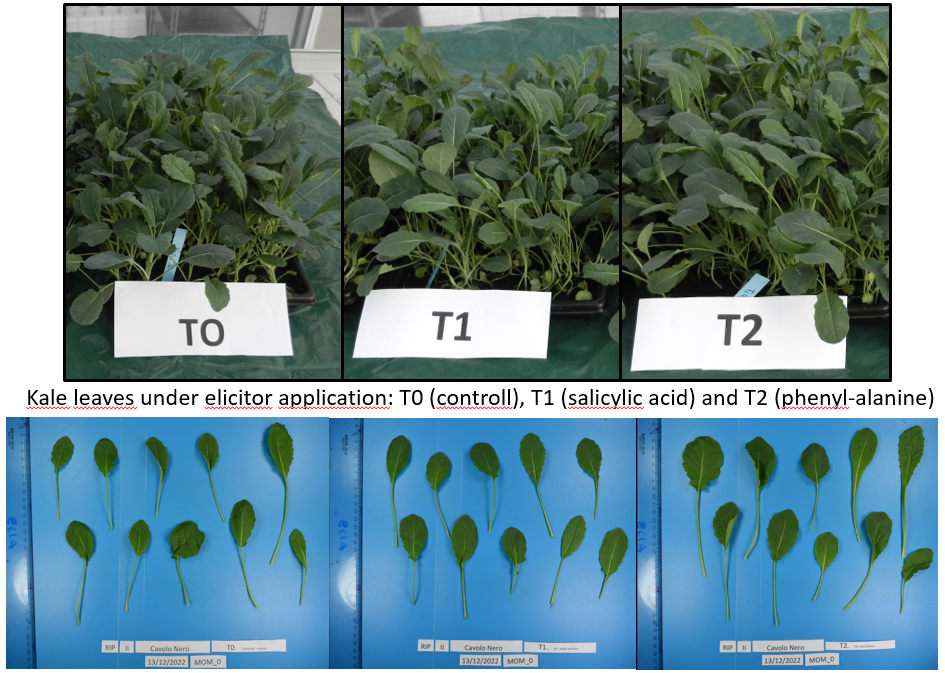

Supplement: Supplementary file 1 [file Image1.png]
